# Systematic Profiling Reveals Propensity of Human Ion Channels for Liquid–Liquid Phase Separation

**DOI:** 10.3390/ijms27198601

**Published:** 2026-09-25

**Authors:** Jiamin Li, Yijing Yao, Ran Yu, Siyu Cui, Yun Dong, Yaqing Lu, Ximing Chen, Longkai Yang, Xin Liu, Ning Wang

**Affiliations:** 1 Department of Pharmacology (State Key Laboratory of Frigid Zone Cardiovascular Diseases (SKLFZCD)), College of Pharmacy, Harbin Medical University, Harbin 150081, China; 17861203305@163.com (Y.Y.); 13844311038@163.com (R.Y.); cuisy2024@163.com (S.C.); yun200105@126.com (Y.D.); 18800418407@163.com (Y.L.); 18222386390@163.com (X.C.); 17865436353@163.com (L.Y.); lx200252@126.com (X.L.); 2 State-Province Key Laboratories of Biomedicine-Pharmaceutics of China and Key Laboratory of Cardiovascular Medicine Research, Ministry of Education, Department of Pharmacology, College of Pharmacy, Harbin Medical University, Harbin 150081, China

**Keywords:** ion channel, LLPS, PS-Self propensity protein, PS-Part propensity protein

## Abstract

Liquid–liquid phase separation (LLPS), a fundamental biophysical process driven by multivalent weak interactions, regulates diverse cellular physiological functions. Ion channels are critical transmembrane protein complexes governing electrophysiological signaling, yet their phase separation characteristics remain largely uncharacterized. Here, we systematically assessed the phase-separation propensity of 140 human ion-channel proteins from the PhaSePred database, and 69 human ion channel proteins were predicted to have a high propensity for LLPS (prediction score ≥ 0.5). Based on the self-assembling phase-separating (PS-Self) and partner-dependent phase-separating (PS-Part) scores of the proteins, we classified ion channel proteins with a ratio (PS-Self/PS-Part) > 1 into the PS-Self group and those with a ratio < 1 into the PS-Part group. Ultimately, 37 proteins were predicted to tend toward PS-Self, and 32 to tend toward PS-Part. Moreover, PS-Self candidates exhibited higher hydropathy and pi–pi contact scores, whereas predicted PS-Part candidates showed higher IDR, LCR, and phosphorylation-associated scores. Experimental analyses identified intracellular regions of Ca_V_1.2, Ca_V_1.3, Na_V_1.5, and K_Ca_2.2 that formed dynamic condensate-like assemblies when expressed in HeLa cells. Our findings elucidate the phase separation classification and core structural domains mediating LLPS in key ion channels and providing a molecular basis for understanding the pathophysiological roles of ion channel phase separation in cardiac and neurological disorders and may inform the development of phase-separation-targeted diagnostic and therapeutic strategies.

## 1. Introduction

### 1.1. The Basic Functions and Physiological Significance of Ion Channels

Ion channels are transmembrane protein complexes that mediate the selective transport of specific ions across cell membranes. They are essential for maintaining resting membrane potential, generating action potentials, transducing cellular signals, and regulating cell volume and osmotic homeostasis [1]. Ion channel dysfunction contributes to various diseases, such as cardiac arrhythmias [2], epilepsy [3], and neurodegenerative disorders [4]. According to ion selectivity, ion channels are mainly classified into sodium, potassium, calcium, non-selective cation (e.g., TRP), and chloride channels.

Among these, voltage-gated sodium (Na^+^), potassium (K^+^), and calcium (Ca^2+^) channels are pivotal for cellular excitability, Na^+^ channels consist of pore-forming α-subunits (*SCN* genes, Na_V_1.1–1.9) and auxiliary β-subunits (key subtypes Na_V_1.5 [cardiac], Na_V_1.1/1.7 [neuronal]) [5]. K^+^ channels [6], the largest family (*KCN* genes) include voltage-gated (K_V_) [7], inward-rectifier (Kir), Ca^2+^-activated (K_Ca_) [8], and two-pore-domain (K_2_P) tetrameric α-subunit subtypes with diverse transmembrane structures. Ca^2+^ channels, also known as voltage-gated calcium channels (VGCCs) comprise α1-subunits (encoded by *CACNA1* genes) divided into high-voltage-activated (HVA; L/P/Q/N/R-type) and low-voltage-activated (LVA (T-type) families paired with β/α2δ/γ auxiliary subunits (critical isoforms Ca_V_1.2 [cardiac], Ca_V_2.1 [neuronal]) [9]. Physiologically, these channels act synergistically—Na^+^ channels initiate action potentials via rapid depolarization [10], K^+^ channels mediate repolarization and stabilize resting membrane potential [11], and Ca^2+^ channels drive excitation-contraction coupling, neurotransmitter release, and pacemaking [12]. Pathologically, genetic mutations acquired dysfunction (e.g., due to ischemia, toxins, or inflammation) cause channelopathies [13] (Na^+^: Brugada syndrome [14], Dravet syndrome, chronic pain; K^+^: long QT syndrome [15]; Ca^2+^: Timothy syndrome, familial hemiplegic migraine [16], absence epilepsy) and excessive Ca^2+^ influx or impaired ion balance contributes to neurodegeneration, heart failure, and excitotoxic injury, making these channels key therapeutic targets for excitable tissue disorders.

### 1.2. Discovery and Biological Significance of Phase Separation Phenomenon

Liquid–liquid phase separation (LLPS) is a biophysical process [17] that drives the spontaneous organization of biomacromolecules into membraneless condensates [18,19]. Dysregulation of phase-separated condensates has also been increasingly associated with diverse human diseases [20]. This phenomenon underpins the formation of subcellular compartments and has emerged as a key regulatory mechanism in diverse physiological functions, including transcriptional control via super-enhancer assembly. By offering a new framework for understanding cellular organization, LLPS has become a pivotal research focus in fields [21] such as oncology [22,23], virology, and neurodegeneration [24].

Bioinformatic analyses have revealed significant enrichment of post-translational modification (PTM) sites within intrinsically disordered regions (IDRs), highlighting PTMs as crucial regulators of phase separation dynamics [25]. Liu et al. discovered that the deubiquitinase USP42 undergoes nuclear LLPS to mediate PLRG1 ubiquitination and phase separation, facilitating nuclear speckle formation and alternative splicing regulation and demonstrating clinical relevance for non-small cell lung cancer prognosis [26].

While these advances have established IDR-mediated [27] condensation as a key mechanism regulating the phase separation of transcriptional regulators and their spatial organization, the LLPS behavior of ion channel proteins remains largely unexplored, representing an important frontier for future investigation. LLPS propensity is influenced by multiple factors, including prion-like regions [28], pi–pi contact [29], IDRs [30], hydropathy [31], coiled-coil regions [32], and low-complexity regions (LCRs) [33]. PhaSePred (version 1.0) is an integrated machine-learning platform that combines multiple established predictors and sequence-related features, including catGRANULE, PLAAC, PScore, ESpritz, SEG, and DeepCoil to evaluate protein phase-separation propensity. Its computational framework, model construction, training, and validation have been described in detail by Chen et al. [34]. Based on the presence of phase-separation-associated sequence features in many ion-channel proteins, we hypothesized that ion channels may harbor intracellular regions with the potential to form biomolecular condensates.

This LLPS phenomenon may represent a novel regulatory mechanism enabling rapid cellular adaptation to environmental stimuli [35] or functional modulation of excitable cells (e.g., neurons and myocytes). Furthermore, this process may contribute to the pathogenic aggregation of disease-associated ion channel proteins (including voltage-gated sodium channels, calcium channels, and potassium channels), offering new perspectives for elucidating related pathophysiological mechanisms and developing targeted therapeutic interventions.

## 2. Results

### 2.1. Factors Affecting LLPS of Ion Channel Proteins

Ion channels are a class of transmembrane proteins embedded in the cell membrane, and their phase separation may affect their electrophysiological functions. Firstly, we selected 140 ion channel protein subtypes derived from humans, including cross-species subtypes, with 125 subtypes in mice and 120 subtypes in rats (Figure 1A, Supplementary Dataset S1). In human ion channel proteins, potassium channels were the most numerous, totaling 100, accounting for 71.4%; calcium channels totaled 26, accounting for 18.6%; and sodium channels totaled 14, accounting for 10.0% (Figure 1B). The likelihood of LLPS is influenced by multiple factors, including prion-like region, pi–pi contact, IDR, hydropathy, coiled-coil region, LCR, and phosphorylation frequency. To assess the LLPS potential of human ion channel proteins, we systematically scored the aforementioned 140 proteins using the predictive algorithm for influencing factors from the catGRANULE database [36]. The results showed that 69 proteins had an LLPS prediction score ≥ 0.5, while 71 proteins scored < 0.5 (Supplementary Dataset S2). In accordance with the scoring criteria of the database and previously studies, a score ≥ 0.5 was defined as the high-propensity threshold for LLPS, indicating that proteins within this score range are more likely to undergo liquid–liquid phase separation. The top 15 ion channel proteins include Ca_V_γ8, Ca_V_2.1, K_V_β3, K_V_7.3, Ca_V_2.2, K_V_3.3, K_V_7.2, Ca_V_α2δ1, Ca_V_3.2, Ca_V_3.3, Ca_V_1.4, Ca_V_β1, Ca_V_1.3, Ca_V_2.3, and Na_V_1.6 (Figure 1C). Although potassium-channel-related proteins represented the largest absolute number of high-scoring candidates (38 proteins), this reflected the larger size of the potassium-channel set. When normalized to family size, high-propensity candidates accounted for 38.0% of potassium-channel-related proteins (38/100), 76.9% of calcium-channel-related proteins (20/26), and 78.6% of sodium-channel-related proteins (11/14). The overall distribution differed significantly among the three channel families (χ^2^ = 17.845, df = 2, *p* = 0.00013; Figure 1D).

### 2.2. Classification of Ion Channel Proteins into Those with a High Propensity for PS-Self and Those with a High Propensity for PS-Part

Protein liquid–liquid phase separation of biomacromolecules can occur as either self-assembling phase-separating (PS-Self) or partner-dependent phase-separating (PS-Part), depending on a combination of various factors. These primarily include external environmental conditions (such as protein concentration, temperature, pH, and ionic strength [37]), as well as the internal structure of protein molecules and multivalent interactions between them; examples of such interactions involve IDR, LCR, electrostatic forces, hydrogen bonding, pi–pi contact, hydrophobic effects, post-translational modification of proteins (PTM). The 69 high-propensity candidates were further evaluated using the PhaSePred PS-Self/PS-Part prediction framework. Proteins with a PS-Self/PS-Part score ratio > 1 were classified as predicted PS-Self candidates, whereas those with a ratio < 1 were classified as predicted PS-Part candidates. This set included 37 PS-Self propensity ion channel proteins and 32 PS-Part propensity ion channel proteins (Figure 2A,B, Supplementary Dataset S3). We systematically assessed the PS-Self and PS-Part potentials of ion channel proteins using the following seven metrics: prion-like region (represented by PLAAC scores [38]), IDR (predicted by the ESpritz algorithm [39]), LCR (scored with the SEG algorithm), and coiled-coil region (quantified with the DeepCoil algorithm [40]). PScore to illustrate multivalent interactions including hydrophobicity, and pi–pi contact. Apart from amino acid sequence composition, features such as PTM frequencies can also provide information in screening PS proteins. Here, we chose phosphorylation (Phos) as the representative PTM type (recorded in PhosphoSitePlus [41]). Because several of these descriptors contribute to the computational prediction framework, their between-group differences are interpreted here as model-associated feature profiles rather than independent validation of the PS-Self/PS-Part classification. The heatmap in Figure 2C therefore displays the corresponding feature scores. Consistent with the original model-associated profile, predicted PS-Self candidates showed higher hydropathy and pi–pi contact scores, whereas predicted PS-Part candidates showed higher IDR, LCR, and phosphorylation-associated scores (Figure 2D–J; Supplementary Dataset S4). To further characterize sequence features potentially associated with phase-separation behavior, we analyzed three additional sequence-level descriptors: charge patterning (κ), aromatic sticker density, and mean inter-sticker distance (Figure 2K). No significant between-group differences were detected for κ, aromatic sticker density, or mean inter-sticker distance (Figure 2L–N).

### 2.3. Ca_V_1.2 Residues 1689–2139 Form Dynamic Condensate-like Assemblies

Based on the structural characteristics of Ca_V_1.2, Ca_V_1.2 was divided into five structural segments to observe the formation of condensates after each domain was transfected (Figure 3A,B). Next, we synthesized GFP-tagged constructs for the five sequences (EGFP-Ca_V_1.2_1-111_, EGFP-Ca_V_1.2_112-782_, EGFP-Ca_V_1.2_783-887_, EGFP-Ca_V_1.2_888-1688_, and EGFP-Ca_V_1.2_1689-2139_) and transfected them into HeLa cells. We observed that overexpression of EGFP-Ca_V_1.2_1689-2139_ resulted in the formation of condensates, with the protein expressed from this fragment exhibiting a punctate green fluorescence pattern that was exclusively localized in the nucleus (Figure 3C). Subsequently, when we added a 1,6-hexanediol to the EGFP-Ca_V_1.2_1689-2139_ overexpression cells, the punctate aggregation of EGFP-Ca_V_1.2_1689-2139_ was completely disrupted, resulting in a diffuse distribution throughout the nucleus (Figure 3D). Because 1,6-hexanediol is a nonspecific perturbant of weak molecular interactions, this observation is considered supportive rather than definitive evidence of liquid-like condensation. Live-cell time-lapse imaging analysis revealed that EGFP-Ca_V_1.2_1689-2139_ in HeLa cells was dynamic and could spontaneously fuse within the cells (Figure 3E). Three-dimensional imaging made the observed aggregates more intuitive and clear (Figure 3F). To further evaluate the phase separation capability of EGFP-Ca_V_1.2_1689-2139_, we examined the fluidity of its protein droplets using confocal fluorescence recovery after conventional photobleaching (FRAP), which allowed us to confirm the dynamic nature of EGFP-Ca_V_1.2_1689-2139_ protein droplets. As shown in Figure 3G, after EGFP-Ca_V_1.2_1689-2139_ protein droplets were photobleached with a strong laser, the fluorescence of the protein droplets recovered to pre-bleach intensity within just a few tens of seconds, indicating that EGFP-Ca_V_1.2_1689-2139_ has good droplet fluidity.

### 2.4. Ca_V_1.3 Residues 1644–2144 Form Dynamic Condensate-like Assemblies

Similarly, Ca_V_1.3 was divided into seven structural segments (EGFP-Ca_V_1.3_1-113_, EGFP-Ca_V_1.3_114-447_, EGFP-Ca_V_1.3_448-519_, EGFP-Ca_V_1.3_520-808_, EGFP-Ca_V_1.3_808-893_, EGFP-Ca_V_1.3_894-1643_, and EGFP-Ca_V_1.3_1644-2144_) to observe the formation of condensates after transfection of each construct. We observed that Ca_V_1.3_1644-2144_ showed the most significant condensate formation. We then used the PhaSePred database to comprehensively assess the liquid–liquid phase separation (LLPS) tendency of Ca_V_1.3, (Figure 4A,B). Next, we synthesized this sequence, constructed an EGFP-tagged Ca_V_1.3_1644-2144_ overexpression plasmid (EGFP-Ca_V_1.3_1644-2144_) and transfected it into Hela cells. We found that the synthesized protein exhibited punctate green fluorescence, completely localized in the nucleus (Figure 4C). Subsequently, when we added a 1,6-hexanediol to the EGFP-Ca_V_1.3_1644-2144_ overexpression cells, the punctate aggregation of EGFP-Ca_V_1.3_1644-2144_ was completely disrupted, resulting in a diffuse distribution throughout the nucleus (Figure 4D). Live-cell time-lapse imaging analysis revealed that EGFP-Ca_V_1.3_1644-2144_ in HeLa cells was dynamic and could spontaneously fuse within the cells (Figure 4E). Three-dimensional imaging made the observed aggregates more intuitive and clear (Figure 4F). To further evaluate the phase separation capability of EGFP-Ca_V_1.3_1644-2144_, we examined the fluidity of its protein droplets using confocal fluorescence recovery after photobleaching (FRAP), which allowed us to confirm the dynamic nature of EGFP-Ca_V_1.3_1644-2144_ protein droplets. As shown in Figure 4G, after EGFP-Ca_V_1.3_1644-2144_ protein droplets were photobleached with a strong laser, the fluorescence of the protein droplets recovered to pre-bleach intensity within just a few tens of seconds, indicating that EGFP-Ca_V_1.3_1644-2144_ has good droplet fluidity.

### 2.5. Na_V_1.5 Residues 1–452 Form Dynamic Condensate-like Assemblies

Na_V_1.5 was divided into five structural segments (EGFP-Na_V_1.5_1-452_, EGFP-Na_V_1.5_453-655_, EGFP-Na_V_1.5_656-986_, EGFP-Na_V_1.5_987-1188_, and EGFP-Na_V_1.5_1189-2020_) to observe the formation of condensates after transfection of each domain. It was observed that Na_V_1.5_1-452_ exhibited the most prominent condensate formation. We then performed a comprehensive evaluation of the LLPS phenomenon of Na_V_1.5 using the PhaSePred database (Figure 5A,B). Next, we synthesized this sequence and constructed an EGFP-tagged Na_V_1.5_1-452_ overexpression plasmid of (EGFP-Na_V_1.5_1-452_), which was transfected into HeLa cells. We found that the protein expressed from this fragment displayed a punctate green fluorescent distribution localized entirely in the cytoplasm (Figure 5C). Subsequently, when we added a 1,6-hexanediol to the EGFP-Na_V_1.5_1-452_ overexpression cells, the punctate aggregation of EGFP-Na_V_1.5_1-452_ was completely disrupted, resulting in a diffuse distribution throughout the cytoplasm. (Figure 5D). Live-cell time-lapse imaging analysis revealed that EGFP-Na_V_1.5_1-452_ in HeLa cells was dynamic and could spontaneously fuse within the cells (Figure 5E). Three-dimensional imaging made the observed aggregates more intuitive and clear (Figure 5F). To further evaluate the phase separation capability of EGFP-Na_V_1.5_1-452_, we examined the fluidity of its protein droplets using confocal fluorescence recovery after photobleaching (FRAP), which allowed us to confirm the dynamic nature of EGFP-Na_V_1.5_1-452_ protein droplets. As shown in Figure 5G, after EGFP-Na_V_1.5_1-452_ protein droplets were photobleached with a strong laser, the fluorescence of the protein droplets recovered to pre-bleach intensity within just a few tens of seconds, indicating that EGFP-Na_V_1.5_1-452_ has good droplet fluidity.

### 2.6. K_Ca_2.2 Residues 1–376 Form Dynamic Condensate-like Assemblies

Based on the structural characteristics of its amino acids, we divided K_Ca_2.2 into two segments: a disordered low-complexity region (residues 1-376) and a calmodulin-binding region (residues 377-849) (Figure 6A,B). We synthesized GFP-tagged constructs for the two sequences (EGFP-K_Ca_2.2_1-376_ and EGFP-K_Ca_2.2_377-849_) and transfected them into HeLa cells. We observed that overexpression of EGFP-K_Ca_2.2_1-376_ resulted in the formation of condensates, with the protein expressed from this fragment exhibiting a punctate green fluorescence pattern (Figure 6C). Subsequently, when we added 1,6-hexanediol, which is used to dissolve phase-separated condensates, to the EGFP-K_Ca_2.2_1-376_ overexpression cells, the punctate aggregation of K_Ca_2.2_1-376_ was completely disrupted, resulting in a diffuse distribution throughout the cell (Figure 6D). Live-cell time-lapse imaging analysis revealed that EGFP-K_Ca_2.2_1-376_ in HeLa cells was dynamic and could spontaneously fuse within the cells (Figure 6E). Three-dimensional imaging made the observed aggregates more intuitive and clear (Figure 6F). To further evaluate the phase separation capability of EGFP-K_Ca_2.2_1-376_, we examined the fluidity of its protein droplets using confocal fluorescence recovery after photobleaching (FRAP), which allowed us to confirm the dynamic nature of K_Ca_2.2_1–376_ protein droplets. As shown in Figure 6G, after EGFP-K_Ca_2.2_1-376_ protein droplets were photobleached with a strong laser, the fluorescence of the protein droplets recovered to pre-bleach intensity within just a few tens of seconds, indicating that K_Ca_2.2_1-376_ has good droplet fluidity.

## 3. Discussion

Liquid–liquid phase separation has emerged as a pivotal regulatory mechanism orchestrating the spatial organization and functional dynamics of biomacromolecules in membraneless cellular compartments, with profound implications for transcriptional regulation, signal transduction, and disease pathogenesis [21]. While LLPS of transcriptional regulators, RNA-binding proteins and viral factors has been extensively characterized [42], the phase separation properties of ion channels—core mediators of cellular electrophysiology—have remained an uncharted frontier in LLPS research. This study systematically evaluated the potential phase separation sequence features of human ion channel proteins based on data from the PhaSePred database [34]. Among 140 human ion channel-related proteins, 69 were found to exhibit a high phase separation propensity, of which 37 showed a high PS-Self propensity and 32 showed a high PS-Part propensity. On this basis, we further assessed the phase separation capacity of four PS-Self candidate ion channel proteins and found that specific fragments of Ca_V_1.2, Ca_V_1.3, Na_V_1.5, and K_Ca_2.2 were able to spontaneously form condensates with dynamic characteristics in cells. These results suggest that ion channel proteins possess the potential to undergo phase separation and may participate in the onset and progression of diseases by regulating protein synthesis, localization, and function.

This study aimed to evaluate the sequence features of ion channel proteins with a high phase separation propensity during spontaneous and non-spontaneous phase separation processes. We calculated the ratio of the predicted PS-Self score to the predicted PS-Part score. A ratio of 1 serves as a natural neutral boundary: a value of 1 indicates that the two mechanisms (PS-Self score and PS-Part score) have comparable scores, a value greater than 1 indicates that the PS-Self propensity is stronger than the PS-Part propensity, and vice versa. We classified ion channel proteins with a ratio greater than 1 into the PS-Self group, and those with a ratio less than 1 into the PS-Part group. This rule is essentially a simplified ratio-based decision/likelihood-ratio framework that does not depend on the absolute magnitude of the scores but only assesses the propensity for phase separation to occur. This yielded 37 proteins with a predicted tendency toward PS-Self and 32 with a predicted tendency toward PS-Part.

After this classification step, we compared several PhaSePred-derived sequence features between the two predicted groups, including prion-like propensity, pi-pi interaction propensity, intrinsic disorder, hydropathy, coiled-coil propensity, low-complexity characteristics, phosphorylation-related features, charge patterning, and sticker-spacers. The results showed that PS-Self candidates had higher hydropathy and pi–pi contact scores, whereas predicted PS-Part candidates showed higher IDR, LCR, and phosphorylation-associated scores. Therefore, we think that the aggregation tendency of ion channel proteins may not be determined by a single sequence feature but is more likely influenced by a combination of multiple sequence properties, structural context, and molecular interactions. Therefore, the PS-Self tendency and PS-Part tendency are better suited as a computational framework for describing and screening potential phase separation behaviors, rather than being interpreted as firmly established biological categories that have been fully validated experimentally.

The intracellular segments of ion channels observed in this study are not necessarily solely experimental products derived from artificially constructed systems. The majority of full-length ion channel proteins are transmembrane proteins, primarily localized on the cell membrane surface [1], such as Na_V_1.5 [43], Ca_V_1.2 [44], and Ca_V_1.3 [45]. According to current literature, no characteristic aggregates can be observed on the cell membrane. However, under specific pathological conditions, ion channel proteins may undergo proteolytic cleavage, giving rise to truncated fragments. Although these fragments do not inherently exhibit phase-separation propensity in the full-length protein, once released, they can aggregate via their intrinsic sequences prone to phase separation and enter the nucleus to exert transcriptional regulatory functions. Previous studies have demonstrated that certain ion channels can generate endogenous fragments with distinct subcellular localization and function. For example, Ca_V_1.2 can generate an endogenous C-terminal fragment (CCAT), which can enter the nucleus and participate in transcriptional regulation; this fragment also exhibits a punctate distribution within the nucleus in primary neurons [46]. Similarly, the C-terminal region of Ca_V_1.3 can be detected in the nuclei of both neonatal and adult atrial myocytes and possesses an independent transcriptional regulatory function [47]. Recent studies on hERG1b further indicate that local fragments derived from ion channels and intact membrane-associated proteins may exhibit distinct molecular assembly behaviors: the N-terminal fragment can form aggregates with dynamic FRAP recovery characteristics, whereas the intact membrane-associated assembly structure does not display the same typical liquid-like behavior [48].

Based on these findings, we propose a potential working model in which, under specific physiological or pathological conditions, ion-channel proteins may undergo proteolytic processing or other forms of structural remodeling, thereby releasing intracellular regions that are otherwise constrained within the intact transmembrane architecture. Once released, these fragments may undergo changes in subcellular localization, local effective concentration, and the molecular interaction environment, which could in turn enable their latent multivalent interaction capacity to become functionally relevant. For certain ion channels, such intracellular fragments may further translocate to the nucleus and participate in transcriptional regulation or other noncanonical ion-channel functions. Therefore, the study of ion-channel phase separation may extend beyond the spatial organization of membrane-embedded channels themselves and encompass intracellular or even nuclear regulatory processes mediated by channel-derived fragments. Nevertheless, this concept remains a working model based on the present findings and existing literature. The current study does not establish whether these specific fragments are endogenously generated under defined disease conditions, nor does it determine whether they undergo bona fide liquid-liquid phase separation at physiological expression levels. Further validation in disease models and endogenous experimental systems will therefore be required.

Several limitations of the present study should also be acknowledged. The current experiments were primarily based on exogenous expression of intracellular ion-channel fragments. Accordingly, these findings are best interpreted as evidence that the corresponding regions possess the potential to form dynamic condensate-like assemblies, rather than as direct demonstration that they necessarily undergo phase separation under physiological endogenous conditions. In addition, for proteins with a predicted PS-Part propensity, partner dependence has not yet been directly validated in the presence of defined physiologically relevant interaction partners. Future studies incorporating specific binding partners, post-translational modification states, and disease-relevant stimuli will be important for determining whether different ion-channel proteins exhibit distinct condensation tendencies and for elucidating the mechanisms that regulate these behaviors.

Overall, the present study does not demonstrate that full-length ion-channel proteins generally undergo liquid–liquid phase separation. Rather, it identifies previously underappreciated intracellular regions within several ion-channel proteins that display condensation propensity and suggests that different ion channels may exhibit varying degrees of predicted PS-Self or PS-Part tendencies. These findings provide a new perspective for understanding the potential spatial organization and noncanonical functions of ion-channel proteins beyond their classical roles in ion conductance. Future studies are warranted to define the physiological and pathological contexts in which these condensation phenomena occur and to determine their functional relevance to ion-channel regulation and disease pathogenesis.

## 4. Materials and Methods

### 4.1. Bioinformatic Analysis

A total of 140 human sodium, potassium, and calcium channel proteins were included in the analysis. Protein sequences and accession information were obtained from UniProt and are provided in Supplementary Dataset S1. The 140 human ion-channel proteins were screened by their overall LLPS-propensity score was output by the web-based prediction tool, with values ranging from 0 to 1. Phase-separation prediction data were obtained from the PhaSePred platform. The overall phase-separation propensity score for each ion channel protein was directly retrieved from PhaSePred and used for the initial screening, with a score of ≥ 0.5 defined as the operational cutoff for high-propensity candidates. For the 69 selected proteins, the PhaSePred-derived PS-Self propensity and PS-Part propensity scores were further collected. The PS-Self/PS-Part ratio was calculated by dividing the PS-Self score by the PS-Part score for each protein; proteins with a ratio > 1 were classified as predicted PS-Self candidates, whereas those with a ratio < 1 were classified as predicted PS-Part candidates. The sequence-feature scores used for the heatmap in Figure 2, including prion-like propensity, pi-pi interaction propensity, intrinsic disorder, hydropathy, coiled-coil propensity, low-complexity characteristics, and phosphorylation-related features, were also directly obtained from PhaSePred. The detailed computational framework, feature integration, model construction, and score calculation implemented in PhaSePred have been described previously by Chen et al. [34].

In addition, FASTA sequences of the 69 proteins were used to calculate independent sequence-level descriptors. Charge patterning was quantified using the κ parameter according to the framework described by Das and Pappu [49,50]. Aromatic residues F/Y/W were operationally defined as stickers; aromatic sticker density was calculated as the fraction of F/Y/W residues in the sequence and mean inter-sticker distance as the average distance between consecutive aromatic stickers. The conceptual basis of aromatic sticker valence and patterning has been described previously [51].

### 4.2. Plasmid Construction

The following segmented plasmids were constructed: Ca_V_1.2 (EGFP-Ca_V_1.2_1-111_, EGFP-Ca_V_1.2_112-782_, EGFP-Ca_V_1.2_783-887_, EGFP-Ca_V_1.2_888-1688_, and EGFP-Ca_V_1.2_1689-2139_); Ca_V_1.3 (EGFP-Ca_V_1.3_1-113_, EGFP-Ca_V_1.3_114-447_, EGFP-Ca_V_1.3_448-519_, EGFP-Ca_V_1.3_520-808_, EGFP-Ca_V_1.3_808-893_, EGFP-Ca_V_1.3_894-1643_, and EGFP-Ca_V_1.3_1644-2144_); Na_V_1.5 (EGFP-Na_V_1.5_1-452_, EGFP-Na_V_1.5_453-655_, EGFP-Na_V_1.5_656-986_, EGFP-Na_V_1.5_987-1188_, and EGFP-Na_V_1.5_1189-2020_); K_Ca_2.2 (EGFP-K_Ca_2.2_1-376_ and EGFP-K_Ca_2.2_377-849_). These plasmids were driven by the CMV promoter and carry an EGFP tag (Novopro).

Ion-channel fragments were cloned into the pEGFP-C1 vector, with EGFP fused to the N terminus of each fragment. For constructs generated using BglII/BamHI cloning, the EGFP insert junction contained the cloning-derived sequence 5′-TCCGGACTCAGATCT-3′, corresponding to SGLRS; no additional flexible linker was introduced. Construct identity was confirmed by sequence verification, with available Sanger sequencing and restriction-digestion/PCR quality-control records. Detailed construct information and plasmid maps are provided in Supplementary Dataset S5.

### 4.3. Culture and Maintenance of Cells

HeLa cells (Human cervical cancer cell line; catalog no. ZQ0068,Zhongqiao Xinzhou Biological Technology; Shanghai, China) were cultured in high glucose DMEM (C11995500BT, Gibco, USA, Grand Island, NY, USA) medium supplemented with 1% penicillin–streptomycin (MA0110, meilunbio, Dalian, China) and 10% fetal bovine serum (04-001-1ACS, Biological Industries, Beit-Haemek, Israel). Cells were grown in a humidified atmosphere with 5% CO_2_ at 37 °C. Cells in all experiments were within 20 passages and free of mycoplasma contamination.

### 4.4. Cell Transfection

HeLa cells were transfected with lipofectamine 2000 (11668-019, Invitrogen, Carlsbad, CA, USA) according to the manufacturer’s protocol. In lipofectamine 2000 transfection, cells were cultured to ~50% confluency in 20 mm glass-bottom dishes (120423EQ01, NEST, Wuxi, China) followed by transfection with 5 μg of plasmids. The cells were changed with fresh DMEM after 6~8 h and incubated for 48 h before further experiments.

### 4.5. Fluorescence Imaging

HeLa cells grown on confocal dishes (120423EQ01, NEST, Wuxi, China) were washed with PBS, then fixed with 4% paraformaldehyde for 15 min and permeabilized with 0.5% Triton X-100 for 30 min. After washing with PBS three times, the cells were blocked with goat serum for 30 min at room temperature. Cells were costained with DAPI (C0065, Solarbio, Beijing, China) to visualize nuclei. Images were taken under a Zeiss LSM 880 (Carl Zeiss Microscopy GmbH, Jena, Germany) fluorescent confocal laser scanning microscope system. Speckle size was determined through ImageJ version 1.50i (National Institutes of Health, Bethesda, MD, USA). Quantitative image analysis, including particle detection and segmentation, has been increasingly applied to extract morphological information from microscopic images [52]. Briefly, the image was loaded into the software, and the scale was established using the “Set Scale” tool. Subsequently, the images were converted to 8-bit, and the threshold was adjusted. Analysis of speckle size was conducted using the “Analyze Particles” tool, whereby the area value of each dot was utilized to indicate the size of individual speckles.

### 4.6. 1,6-Hexanediol Treatment

HeLa cells were transfected with plasmids encoding mEGFP fused indicated proteins for 48 h. For 1, 6-hexanediol treatment, transfected cells were treated with 3% 1,6-hexanediol (G2201258, Aladdin, Shanghai, China) in PBS for 5 min. Images were acquired by confocal fluorescence microscopy after cells were treated.

### 4.7. Live-Cell Imaging

HeLa cells were cultured on the confocal dishes (120423EQ01, NEST, Wuxi, China) and transfected with 5 μg plasmid for 48 h. The cells were washed three times with fresh medium. The cells were maintained in a moisturized environmental chamber (5% CO_2_) on the Zeiss LSM 880 confocal laser scanning microscope platform (Carl Zeiss Microscopy GmbH, Jena, Germany) during live cell imaging.

### 4.8. Fluorescence Recovery After Photobleaching (FRAP)

Cells were grown on glass-bottom dishes until they reached the appropriate density. Cells were transfected with plasmids. After 48 h incubation, a Zeiss LSM 880 confocal microscope system was utilized to conduct cellular FRAP experiments at 37 °C in a live-cell-imaging chamber. GFP signals in regions of interest (ROI) were fully photobleached by using a 488 nm laser on a Zeiss LSM 880 confocal microscope. Fluorescence intensity of ROI between pre-bleached and at the start of recovery after bleaching was recorded by microscope. The prebleached fluorescence intensity was normalized to 1 and the signal after bleaching was normalized to the prebleached level. GraphPad Prism 10 was employed to fit the FRAP data to a single exponential model.

### 4.9. Three-Dimensional Imaging and Analysis

HeLa cells on glass slides transiently transfected with plasmids were fixed using 4% PFA (Solarbio, Beijing, China). Z-stack images were acquired using a Zeiss LSM 880 confocal microscope. Z-stack images were reconstructed in Imaris for three-dimensional visualization.

### 4.10. Statistics Analysis

Images were analyzed with ImageJ. Statistical analyses were performed using GraphPad Prism. Comparisons of sequence-derived features between predicted PS-Self and PS-Part proteins (Figure 2D–J; Figure 2L–N) were performed using a two-tailed Mann-Whitney U test. Differences in the proportions of high-propensity candidates among potassium, calcium, and sodium channel families were assessed using the chi-square test, with pairwise Fisher’s exact tests where appropriate. For experimental comparisons between two independent groups, a two-tailed unpaired *t*-test was used. For experiments involving more than two groups, one-way ANOVA followed by an appropriate multiple-comparison test was used when applicable. Data from cell-based experiments are presented as mean ± SEM unless otherwise indicated. *p* values < 0.05 were considered statistically significant.

## 5. Conclusions

We identify widespread LLPS propensity across human ion channels and show that PS-Self and PS-Part candidates possess distinct sequence architectures. Representative intracellular regions of Ca_V_1.2, Ca_V_1.3, Na_V_1.5, and K_Ca_2.2 form dynamic condensates in cells. These findings provide a focused foundation for future studies of condensate-dependent ion-channel organization and function.

## Figures and Tables

**Figure 1:**
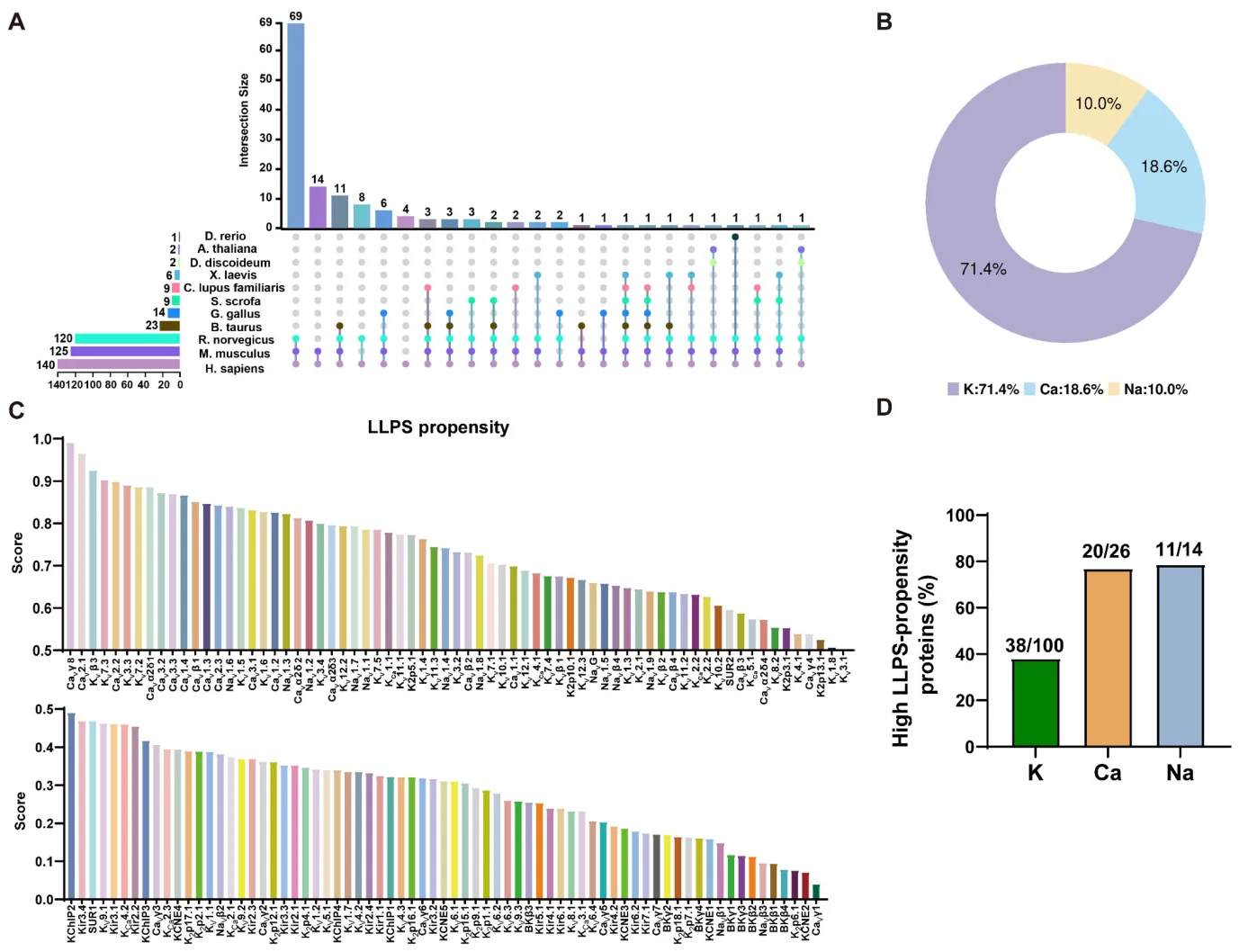
Probability of LLPS occurring in ion channel proteins. (**A**). Distribution of 140 ion channel proteins across different species. (**B**). The proportion of different ion channel proteins. (**C**). Phase separation score of ion channel proteins, LLPS prediction score ≥ 0.5 (up), LLPS prediction score < 0.5 (down). (**D**). Fraction of high-propensity candidates within each channel family.

**Figure 2:**
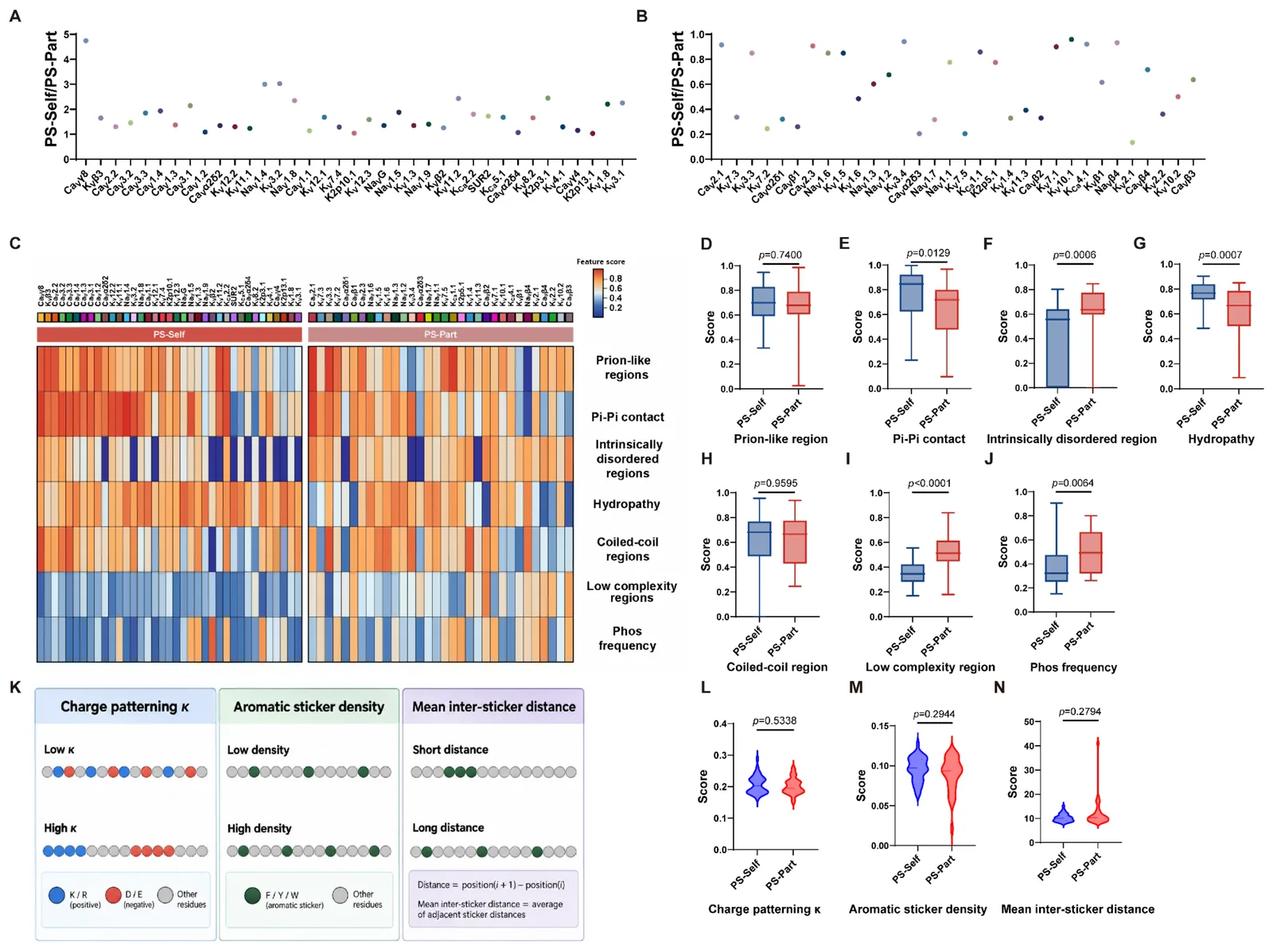
Classification of human ion channel proteins based on PS-Self/PS-Part ratios and comparative analysis of their sequence features. (**A**,**B**). Classification of proteins based on the PS-Self/PS-Part ratio calculated from the PhaSePred database [34]. (**C**). Heatmap illustrating the global profile of seven sequence-based features across 69 ion channel proteins analyzed. (**D**–**J**). Comparison of seven PS-related features between the PS-Self group and PS-Part group. (**K**). Schematic diagram representation of charge patterning κ, aromatic sticker density, and mean inter-sticker distance. (**L**–**N**). Independent sequence-level analyses of charge-patterning κ, aromatic sticker density, and mean inter-sticker distance.

**Figure 3:**
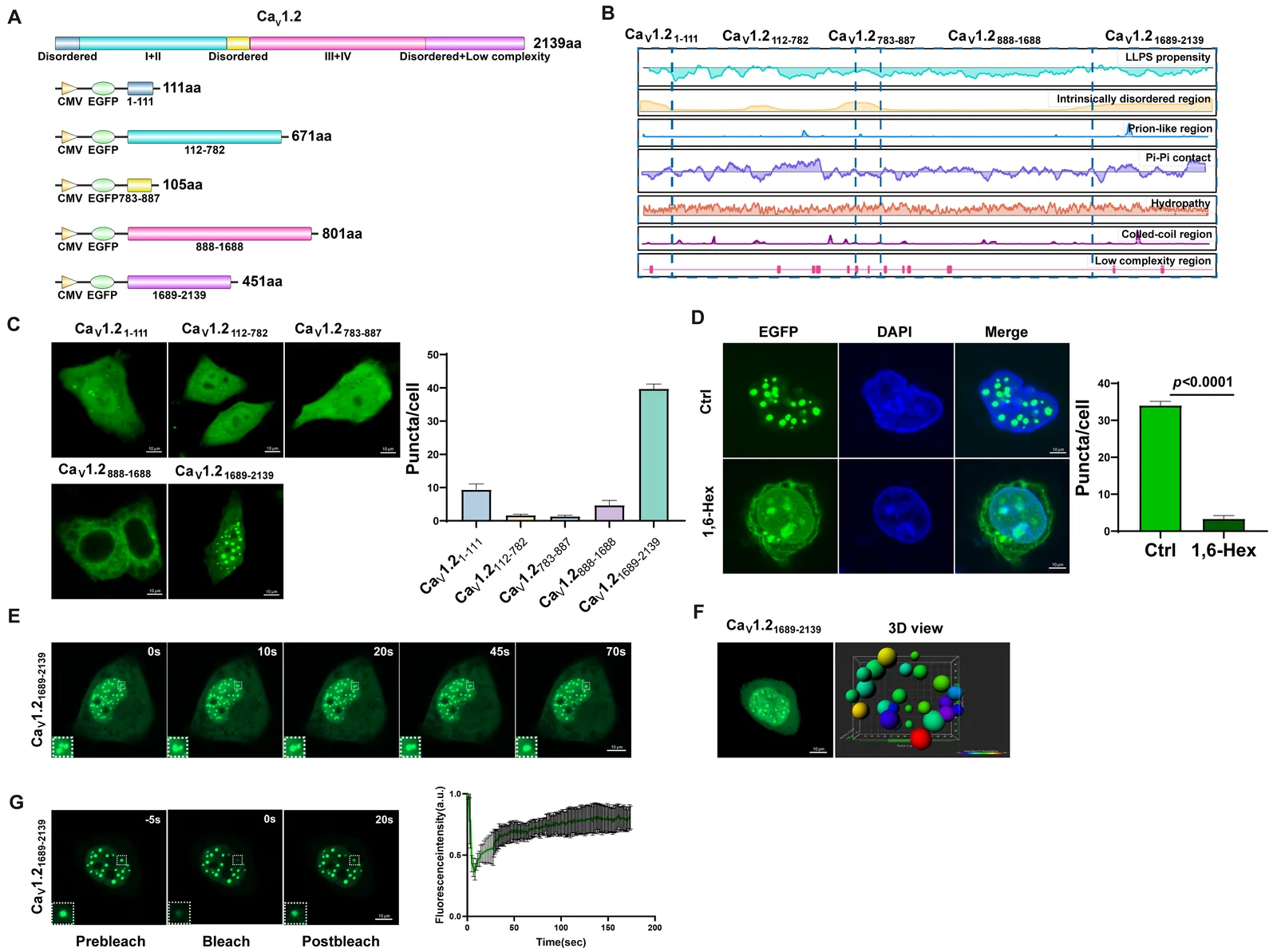
Ca_V_1.2 drives liquid–liquid phase separation via its residues 1689–2139. (**A**). Diagram of Ca_V_1.2 structure segments. (**B**). Comprehensive assessment of Ca_V_1.2 liquid–liquid phase separation phenomenon by PhaSePred. (**C**). Live-cell imaging to observe the distribution of EGFP-Ca_V_1.2 aggregates in cells. Scale bar, 10 μm. (**D**). Fluorescence imaging of Ca_V_1.2_1689-2139_ in HeLa cells (green: Ca_V_1.2_1689-2139;_ blue: DAPI). Scale bar, 10 μm. (**E**). Time-lapse imaging to analyze of the fusion ability of EGFP-Ca_V_1.2_1689-2139_ in cells. Scale bar, 10 μm. (**F**). Three-dimensional reconstruction of EGFP-Ca_V_1.2_1689-2139_ condensates in HeLa cells. (**G**). FRAP to verify the mobility of EGFP-Ca_V_1.2_1689-2139_ in cells. Scale bar, 10 μm.

**Figure 4:**
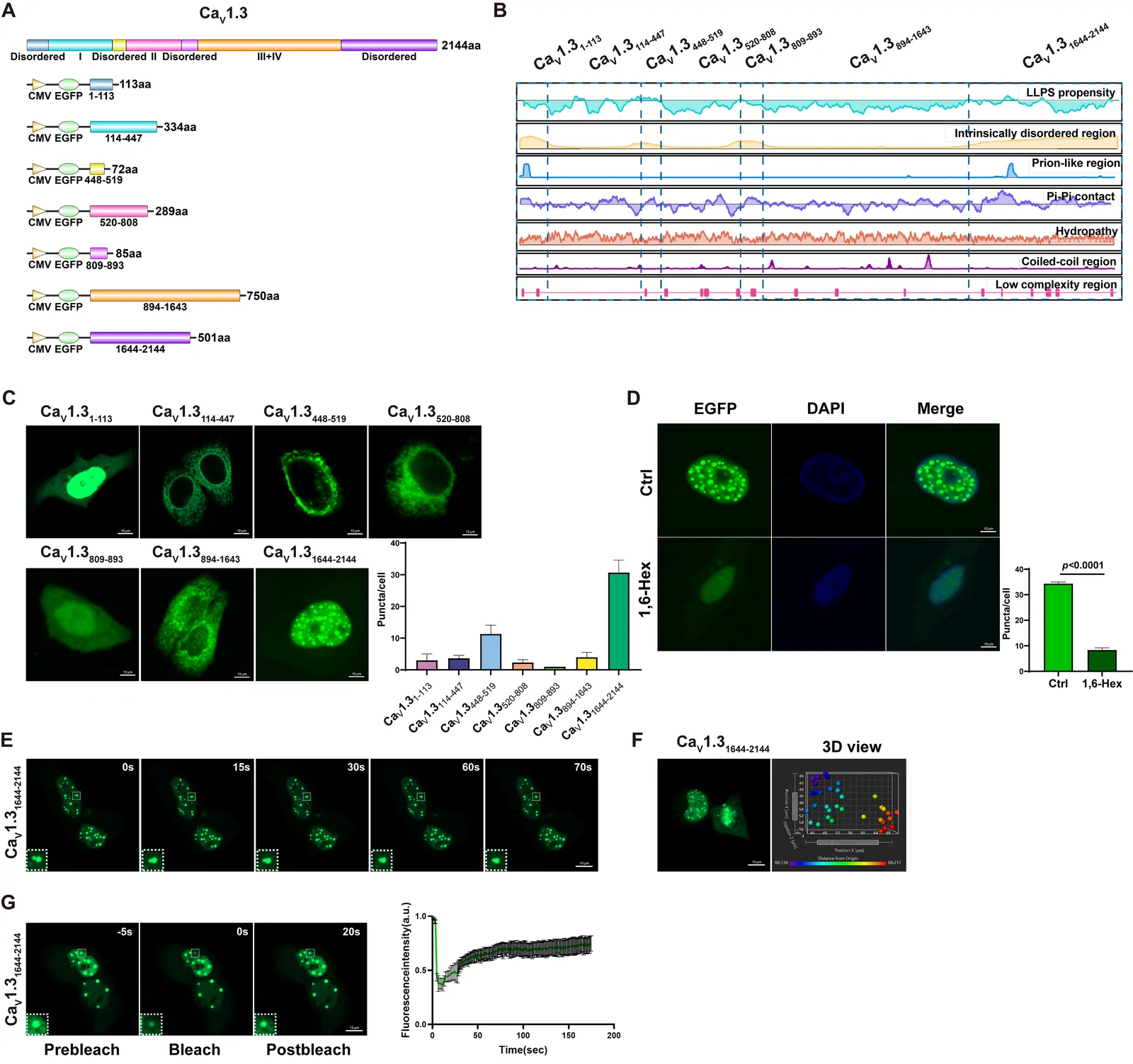
Ca_V_1.3 drives liquid–liquid phase separation via its residues 1644–2144. (**A**). Diagram of Ca_V_1.3 structure segments. (**B**). Comprehensive assessment of Ca_V_1.3 liquid–liquid phase separation phenomenon by PhaSePred. (**C**). Live-cell imaging to observe the distribution of EGFP-Ca_V_1.3 aggregates in cells. Scale bar, 10 μm. (**D**). Fluorescence imaging of Ca_V_1.3_1644-2144_ in HeLa cells (green: Ca_V_1.3_1644-2144;_ blue: DAPI). Scale bar, 10 μm. (**E**). Time-lapse imaging to analyze the fusion ability of EGFP-Ca_V_1.3_1644-2144_ in cells. Scale bar, 10 μm. (**F**). Three-dimensional reconstruction of EGFP-Ca_V_1.3_1644-2144_ condensates in HeLa cells. (**G**). FRAP to verify the mobility of EGFP-Ca_V_1.3_1644-2144_ in cells. Scale bar, 10 μm.

**Figure 5:**
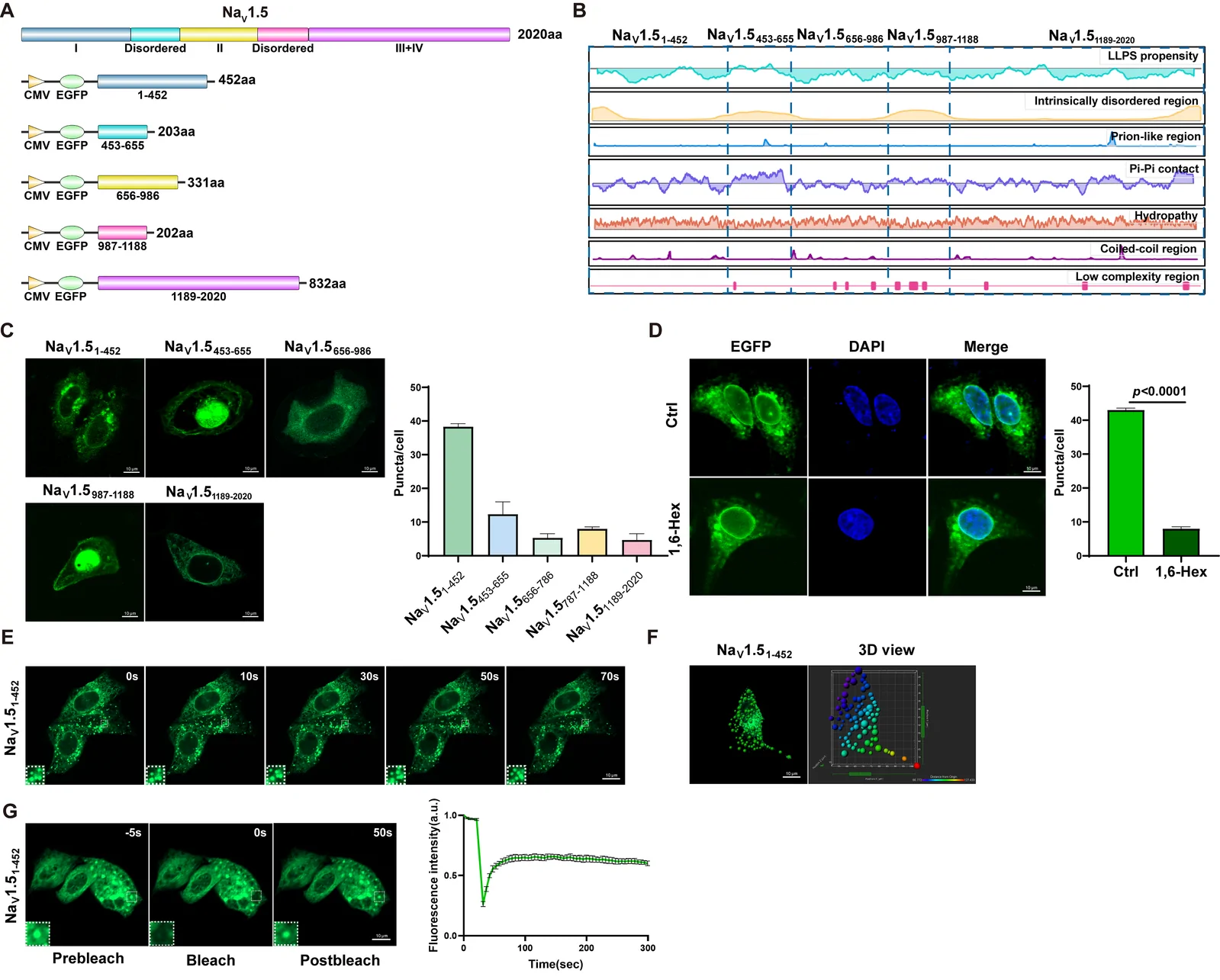
Na_V_1.5 drives liquid–liquid phase separation via its residues 1-452. (**A**). Diagram of Na_V_1.5 structure segments. (**B**). Comprehensive assessment of Na_V_1.5 liquid–liquid phase separation phenomenon by PhaSePred. (**C**). Live-cell imaging to observe the distribution of EGFP-Na_V_1.5 aggregates in cells. Scale bar, 10 μm. (**D**). Fluorescence imaging of Na_V_1.5_1-452_ in HeLa cells (green: Na_V_1.5_1-452;_ blue: DAPI). Scale bar, 10 μm. (**E**). Time-lapse imaging analysis of the fusion ability of EGFP-Na_V_1.5_1-452_ in cells. Scale bar, 10 μm. (**F**). Three-dimensional reconstruction of EGFP-Na_V_1.5_1-452_ condensates in HeLa cells. (**G**). FRAP analysis to verify the mobility of EGFP-Na_V_1.5_1-452_ in cells. Scale bar, 10 μm.

**Figure 6:**
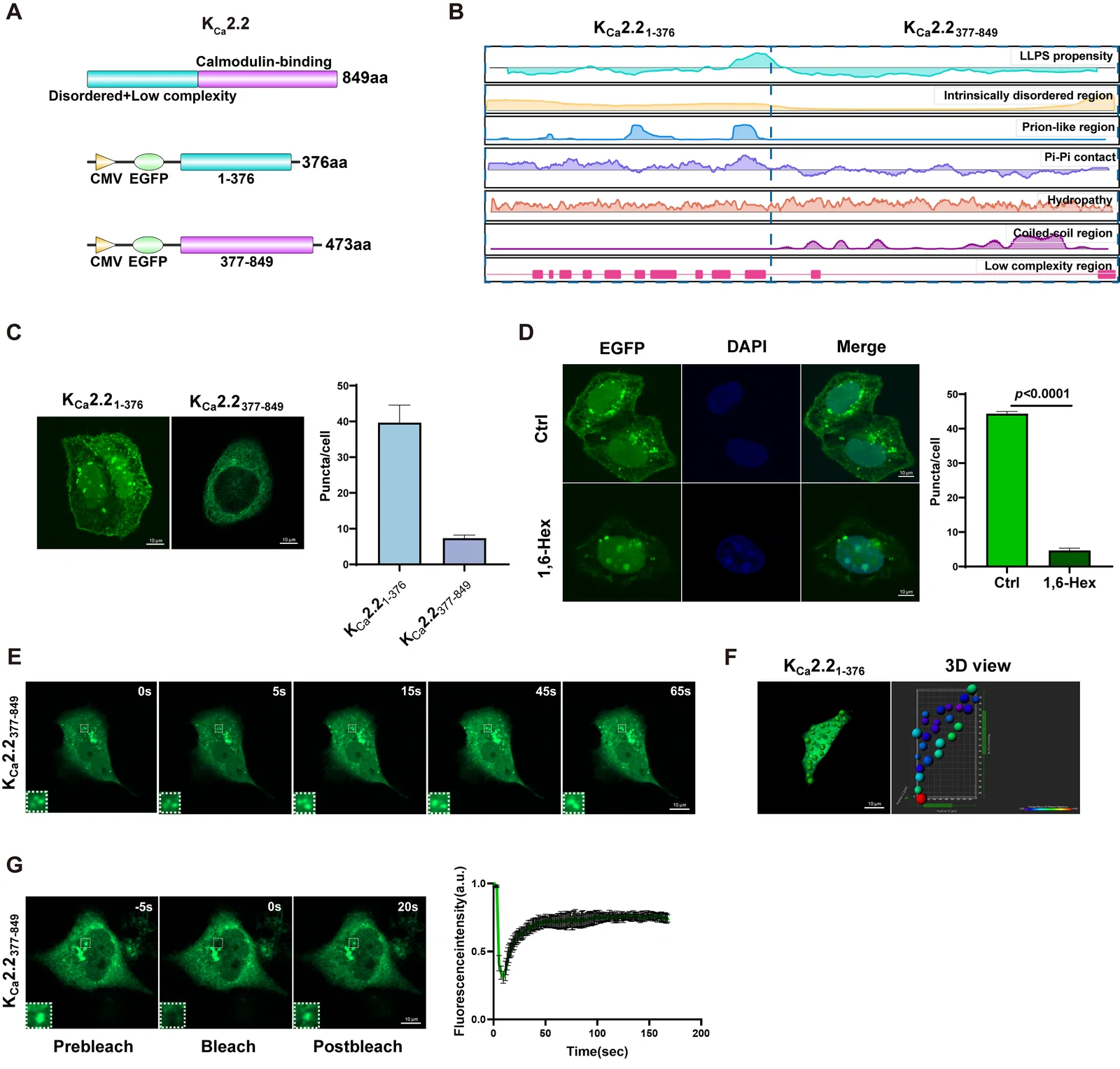
K_Ca_2.2 drives liquid–liquid phase separation via its residues 1–376. (**A**). Diagram of K_Ca_2.2 structure segments. (**B**). Comprehensive assessment of K_Ca_2.2 liquid–liquid phase separation phenomenon by PhaSePred. (**C**). Live-cell imaging to observe the distribution of EGFP-K_Ca_2.2 aggregates in cells. Scale bar, 10 μm. (**D**). Fluorescence imaging of K_Ca_2.2_1-376_ in HeLa cells (green: K_Ca_2.2_1-376;_ blue: DAPI). Scale bar, 10 μm. (**E**). Time-lapse imaging analysis of the fusion ability of EGFP-K_Ca_2.2_1-376_ in cells. Scale bar, 10 μm. (**F**). Three-dimensional reconstruction of EGFP-K_Ca_2.2_1-376_ condensates in HeLa cells. (**G**). FRAP analysis the mobility of EGFP-K_Ca_2.2_1-376_ in cells. Scale bar, 10 μm.

## Data Availability

The protein phase separation prediction data analyzed in this study were obtained from the publicly available PhaSePred prediction server at http://predict.phasep.pro/ (accessed on 25 September 2026). The data generated during this study and supporting the findings of this article are available from the corresponding authors upon reasonable request.

## Supplementary Materials

The following supporting information can be downloaded at: https://www.mdpi.com/article/10.3390/ijms27198601/s1.
